# Hearing voices in the resting brain: A review of intrinsic functional connectivity research on auditory verbal hallucinations

**DOI:** 10.1016/j.neubiorev.2015.04.016

**Published:** 2015-05-05

**Authors:** Ben Alderson-Day, Simon McCarthy-Jones, Charles Fernyhough

**Affiliations:** a Department of Psychology, Durham University, Science Laboratories, South Road, Durham DH1 3LE, United Kingdom; b Department of Cognitive Science, Australian Hearing Hub, Macquarie University, 16 University Avenue, NSW 2109, Australia

**Keywords:** Resting state, Default mode network, Voice-hearing, Inner speech, Schizophrenia

## Abstract

Resting state networks (RSNs) are thought to reflect the intrinsic functional connectivity of brain regions. Alterations to RSNs have been proposed to underpin various kinds of psychopathology, including the occurrence of auditory verbal hallucinations (AVH). This review outlines the main hypotheses linking AVH and the resting state, and assesses the evidence for alterations to intrinsic connectivity provided by studies of resting fMRI in AVH. The influence of hallucinations during data acquisition, medication confounds, and movement are also considered. Despite a large variety of analytic methods and designs being deployed, it is possible to conclude that resting connectivity in the left temporal lobe in general and left superior temporal gyrus in particular are disrupted in AVH. There is also preliminary evidence of atypical connectivity in the default mode network and its interaction with other RSNs. Recommendations for future research include the adoption of a common analysis protocol to allow for more overlapping datasets and replication of intrinsic functional connectivity alterations.

Auditory verbal hallucinations (AVH) refer to hearing voices in the absence of an external stimulus. They are considered a core feature of schizophrenia, presenting in 60–90% of cases (Baethge et al., 2005; Bauer et al., 2011). They can also feature in a range of other psychiatric disorders (such as bipolar disorder and depression) and are experienced without a need for psychiatric care in a minority of the general population (Johns et al., 2014). A number of studies have examined brain activation either during AVH or in relation to hallucination predisposition, but the neural basis of the phenomenon is still not well understood (Allen et al., 2012). In this context, there has been a growing interest in the potential for resting state networks—i.e., the intrinsic organization and spontaneous activation of groups of brain regions—to explain how and why hallucinatory experiences occur (Jardri et al., 2013; Northoff and Qin, 2011).

## 1 Resting state networks and psychopathology

Resting state networks (RSNs) denote groups of brain regions that correlate in their spontaneous activity when idle or ‘at rest’. In fMRI, such spontaneous correlations occur due to low-frequency fluctuations in the hemodynamic response, and are interpreted as evidence of intrinsic, functional connections among brain regions. Intrinsic connectivity can be assessed by selecting a seed region and examining its correlation across time with either specific regions of interest or the whole brain. Alternatively, techniques such as independent components analysis (ICA) can be used to identify networks of areas that tend to covary at rest, without the need for specifying a seed region. Parts of a network can then be assessed for their average connectivity with other regions (i.e., connectivity strength) and the extent to which they mediate other paths within the network (sometimes called ‘betweenness centrality’; Freeman, 1977).

The most well-known resting network is the ‘default mode’ network (DMN; Raichle et al., 2001), a collection of regions including the medial prefrontal cortex, medial temporal lobe, lateral parietal cortex, precuneus, and posterior cingulate. The DMN was originally defined by its tendency to negatively correlate with task-positive activity, prompting the concept of a default state. It is now recognized that the DMN, while often anti-correlated with externally-guided activity, is active in a range of internally-directed cognitive processes, including mind-wandering, autobiographical memory, and future thinking (Buckner et al., 2008). Other notable networks showing intrinsic connectivity at rest include the central executive network (CEN), which links posterior parietal regions to prefrontal cortex (sometimes also referred to as the fronto-parietal network, or FPN), and the salience network (SN), which includes the insula and its connection to the anterior cingulate and the supplementary motor area (Bressler and Menon, 2010). Specific networks underpinning sensory processing are also evident at rest, although these will usually be more apparent during engagement in an externally-driven, task-positive process.

The potential relevance of RSNs to understanding psychopathology in general, and psychosis in particular, has been noted by a number of authors (Broyd et al., 2009; Whitfield-Gabrieli and Ford, 2012; Williamson, 2007). Studies of people with schizophrenia have reported altered connectivity patterns in the DMN and other resting networks, alongside atypical modulation of internally and externally focused attention (Kim et al., 2009; Öngür et al., 2010; Pomarol-Clotet et al., 2008). It is unclear, however, how much schizophrenia case–control studies alone can tell us about the resting state in relation to AVH, for three reasons.

First, schizophrenia is a highly heterogeneous disorder involving widely varying configurations of positive and negative symptoms. Any resting differences observed between patients and controls could relate to AVH, or a number of other symptoms or confounds, including medication. Second, a significant minority of schizophrenia patients do not experience AVH (around 1/3; e.g., Bauer et al., 2011), again limiting any specific conclusions about resting state differences and AVH. Third, most studies of the resting state in schizophrenia have only examined resting networks as if they reflect stable, trait-based markers for psychopathology. AVH, however, are intrinsically transient and unpredictable phenomena; understanding how they might emerge from RSNs requires a close examination of the current hallucination status of participants, the presence of hallucinations during scanning, and the time-course of neural networks prior to hallucination occurring.

This problem is now beginning to be addressed by a growth in the number of studies seeking directly to link resting-state characteristics to the propensity for AVH. In some cases this has involved constraining schizophrenia samples to only include those with AVH, and then comparing them either with healthy controls (Sommer et al., 2012; Vercammen et al., 2010) or healthy controls plus a clinical control group of participants with schizophrenia but no hallucinations (Gavrilescu et al., 2010; Hoffman et al., 2011). Other studies have used mixed schizophrenia groups and specifically reported on correlations between AVH severity and resting-state characteristics (Rotarska-Jagiela et al., 2010; Sorg et al., 2013). Finally, two studies have reported on intrinsic functional connectivity in a sample of people who experience AVH without need for psychiatric care, i.e. ‘non-clinical’ voice-hearers (Diederen et al., 2013; Van Lutterveld et al., 2014).

The following review outlines what can be said so far about the resting state and its role in understanding AVH. Section 2 outlines three main hypotheses about resting state dysfunction and AVH. In Section 3, findings from AVH-specific resting-state studies are reviewed, organized according to the brain areas and networks that have been the primary foci of research. Section 4 considers a selection of methodological confounds to studying intrinsic functional connectivity using fMRI, and examines how they have been addressed by existing studies.

## 2 Resting state hypotheses and auditory verbal hallucinations

As AVH by definition occur in the absence of an external stimulus, there is an immediate plausibility to the idea that an atypical resting state could give rise to a hallucinatory experience. A starting assumption may be that AVH result from abnormally high or atypically moderated resting activity in primary auditory cortices, giving rise to spontaneous internal signals that are misattributed as external, or an over-sensitivity of auditory cortices to top–down expectancy effects (for accounts of this kind, see Cho and Wu, 2013; Hunter et al., 2006; Mintz and Alpert, 1972).

Such hypotheses are supported by evidence of structural changes to primary auditory cortex in schizophrenia patients with AVH (Hubl et al., 2010), and computational models of schizophrenia that posit shallow differences between resting and externally-generated neuronal states (Rolls et al., 2008). However, they are not necessarily supported by neuroimaging evidence of the auditory cortex during AVH. While some symptom-capture studies have observed primary auditory cortical activity during the experience of AVH (Dierks et al., 1999), recent meta analyses have indicated that such activation is not obviously apparent during hallucinations (Jardri et al., 2011; Kühn and Gallinat, 2012). Many studies have instead reported activation of secondary and association auditory areas further up the auditory processing stream. Accordingly, it has been suggested that evidence of primary auditory cortex activity during AVH, where it has been observed, is more likely to reflect a back-propagation of activity from association cortices (Jardri et al., 2013).

A more common result in symptom-capture studies has been the observation of activation in areas associated with speech production, such as the left inferior frontal gyrus (IFG) (Kühn and Gallinat, 2012). Such findings support models of AVH that explain the experience in terms of a failure to monitor one’s own internal speech, resulting in its mistaken attribution to an external source (Feinberg, 1978; Ford et al., 2002; Frith, 1992). Regarding the resting state, such models would predict a pattern of atypical functional connectivity between typical speech processing areas, primarily within a left fronto-temporal network including the IFG and superior and middle temporal gyri, but potentially extending to right hemisphere language ‘homologue’ areas (Sommer et al., 2008). Reduced structural connectivity in the left arcuate fasciculus, which links frontal and temporal language regions, has been found in schizophrenia patients with AVH compared to healthy controls (Geoffroy et al., 2014). More recently this has also been observed for patients with AVH compared to both schizophrenia patients without hallucinations and schizophrenia patients with hallucinations in non-auditory verbal modalities (McCarthy-Jones et al., 2015), supporting a model of disrupted functional interaction within speech-processing systems.

A third hypothesis is that AVH somehow arise from the activity and function of the DMN, which by definition will usually be prominent during rest. In such an account AVH could arise from misattributed productions of the DMN, either via an atypical interaction between the DMN and other networks at rest (Northoff and Qin, 2011), or a failure to maintain the DMN in a stable state (Jardri et al., 2013). The range of functions associated with the DMN—including introspective thought, autobiographical memory and self–other attributions—make it amenable to explaining both the variation in AVHs that people experience and their often personal and self-directed nature (Ffytche and Wible, 2014; Vercammen et al., 2010).

In support of the latter hypothesis, atypical characteristics of the DMN have been found in schizophrenia. Compared to healthy controls, people with schizophrenia have shown reduced suppression of the DMN during task-related periods (Whitfield-Gabrieli et al., 2009), elevated connectivity within the DMN at rest (Zhou et al., 2007), atypical connectivity between DMN hubs (Bluhm et al., 2007), and less independence between the DMN and task-positive networks (see Whitfield-Gabrieli and Ford, 2012, for a review). While these findings point to atypical DMN function in schizophrenia in general, they may also play a role in the development of AVH in particular.

## 3 Resting-state findings in AVH-specific studies

Findings on rest and AVH can be grouped into three clusters: (i) resting connectivity of the auditory cortex and language regions (including the left inferior frontal gyrus and left superior and middle temporal gyri); (ii) areas associated with default mode function, including lateral temporoparietal regions, midline structures, and the hippocampal formation (Buckner et al., 2008), and (iii) other individual areas and networks, including interactions between different RSNs (see Table 1).

### 3.1 Auditory/language-processing regions

#### 3.1.1 Primary auditory cortex

Two studies have specifically examined connectivity of primary auditory cortex (PAC) in AVH. Using a seed-based approach, Gavrilescu et al. (2010) compared resting connectivity between the left and right PAC in patients with schizophrenia and AVH (SzAVH+ henceforth), patients with schizophrenia but no AVH (SzAVH−), and a sample of healthy controls (HC). SzAVH+ participants showed reduced connectivity between left and right hemispheres for both primary and secondary auditory areas compared to the two other groups, leading the authors to propose that interhemispheric connectivity problems may be specific to those with AVH.

Shinn et al. (2013) also compared SzAVH+, SzAVH−, and HC groups, but examined PAC connectivity in relation to the rest of the brain. For left PAC, they observed increased connectivity with the left superior parietal lobule and left middle frontal gyrus, and reduced connectivity to right hippocampal and thalamic regions in SzAVH+. Hallucination severity in this group also positively correlated with the connectivity between left PAC and a range of regions, including left IFG, left STG, the anterior and posterior cingulate cortex, and right orbitofrontal cortex (no other symptom correlations were reported). In contrast, no altered connectivity was found between the right PAC and any other region.

The contrast in findings between these two studies is likely to arise from differences in the kind of analysis deployed. The focus on left–right PAC connectivity by Gavrilescu et al. (2010) may have missed wider connectivity alterations with the rest of the brain. In contrast, Shinn et al.’s (2013) whole-brain analysis could have highlighted specific interhemispheric problems, but did not, even when using the same voxel threshold as Gavrilescu et al. (2010). The lack of atypical connectivity for right PAC and wide range of connectivity alterations for left PAC observed by Shinn et al. (2013) suggest that connectivity differences in AVH are unlikely to primarily reside in bilateral communication of the auditory cortices. Furthermore, the presence of symptom correlations in Shinn et al. (2013) reinforces the suggestion that intrinsic connectivity of the left PAC in particular is related to AVH.

#### 3.1.2 Superior and middle temporal gyri

Four studies have reported on seeds placed in superior and middle temporal gyri (Clos et al., 2014; Diederen et al., 2013; Hoffman et al., 2011; Sommer et al., 2012), while two have reported on connectivity in these areas using network-based methods (Van Lutterveld et al., 2014; Wolf et al., 2011). Hoffman et al. (2011) examined the connectivity of both the left and right posterior superior temporal gyri (STG) to left inferior frontal gyrus in SzAVH+, SzAVH−, and HC individuals. Although no altered connectivity between these two regions was found specific to SzAVH+, greater functional connectivity was found in SzAVH+ compared to both control groups, in a corticostriatal loop involving the IFG, the bilateral posterior STG, and the putamen. Based on evidence of putamen involvement in language initiation (Price, 2010), Hoffman et al. (2011) suggested that this may give rise to an “overabundance of language representations that can become hallucinogenic” in those with AVH (p. 412).

In contrast, Sommer et al. (2012) observed reduced connectivity between left STG and left IFG in a sample of participants with psychosis and AVH compared to controls. Functional connectivity was also lower between left STG and left hippocampus, an effect that was particularly prominent in participants who reported AVH during scanning, and correlated negatively with hallucination severity on the PANSS (Kay et al., 1987). In further analysis of the same sample, Clos et al. (2014) also reported reduced connectivity between a seed in left middle temporal gyrus and the right STG. However, neither study included a comparison with individuals who had psychosis but no AVH, meaning that the observed alterations to connectivity may not be specific to the presence of hallucinations.

One way to avoid such a concern is to study intrinsic connectivity in those with AVH but no psychosis. Diederen et al. (2013) compared connectivity in 25 non-clinical participants with AVH and 25 controls across a selection of seeds that included left and right STG. Elevated connectivity was observed between the two STG sites and the right IFG. Network analysis of an overlapping group of participants by Van Lutterveld et al. (2014) indicated that the left STG in particular showed greater connectivity strength and betweenness centrality in participants with AVH compared to controls. That is, the left STG appeared to act as a more important resting hub for those with AVH, in terms of being more likely to be connected to other brain areas in general. In addition, the right middle temporal gyrus also showed greater levels of connectivity to other areas in AVH participants. The sample reported on in these two studies showed some evidence of sub-clinical characteristics in other domains of psychosis (e.g., unusual or grandiose beliefs), meaning that they should not simply be seen as the equivalent of healthy controls with AVH (Sommer et al., 2010). However, it seems unlikely that such characteristics would explain alterations to resting connectivity when the group was more prominently characterized in terms of proneness to AVH.

The findings of Diederen, van Lutterveld, and colleagues also correspond with evidence from a network analysis in SzAVH+ individuals. Wolf et al. (2011) found that SzAVH+ participants showed increased connectivity strength for left STG and the MTG bilaterally compared to HC. This did not involve a non-hallucinating schizophrenia group, but did include some evidence of symptom correlation: a positive correlation was observed between symptom severity and left STG connectivity that was specific to hallucinations (as measured on the PSYRATS; Haddock et al., 1999) rather than positive symptoms in general (measured on the PANSS).

In sum, there is evidence of atypical connectivity of the STG and MTG from resting-state studies, but results are very mixed. In both clinical and non-clinical participants there is evidence to suggest that the left STG in particular is likely to show elevated connectivity to other brain regions at rest. This is not necessarily the case, however, for connectivity between superior temporal areas and left IFG; in some cases this has been proposed to be intact or possibly elevated in those with AVH (Hoffman et al., 2011), but there is also evidence of reduced STG–IFG functional connectivity (Sommer et al., 2012).

#### 3.1.3 Inferior frontal gyrus

Beyond its coupling with temporal regions, three studies have specifically reported on connectivity in the left inferior frontal gyrus (IFG). As noted above, Hoffman et al. (2011) observed elevated connectivity between left IFG and the putamen in SzAVH+ compared to SzAVH−, which formed part of a corticostriatal loop encompassing language regions and the striatum. In Clos et al. (2014), decreased connectivity for AVH participants was evident between left IFG, left dorsolateral prefrontal cortex, ventrolateral prefrontal cortex, and inferior parietal areas (bilaterally). Alongside this, increased connectivity was observed between left IFG, left insula, and the supplementary motor area, although the lack of an SzAVH− control group limits inferences about the specificity of these changes to AVH. Finally, in non-clinical hallucinating participants, Diederen et al. (2013) observed increased connectivity between left IFG and left parahippocampal cortex, suggesting elevated communication between language regions and areas responsible for mediating memory retrieval.

In addition to the left IFG, the right IFG has also been used as a seed region. Sommer et al. (2012) found that SzAVH+ had increased connectivity with the right parahippocampal gyrus, compared to healthy controls, but reduced connectivity with the right dorsolateral prefrontal cortex (DLPFC). However, the strong association of the right DLPFC with delusions (Coltheart, 2010) highlights that the lack of a SzAVH− control group could have led to these results being confounded by other symptoms associated with schizophrenia. This brings into question their specificity to AVH.

For the inferior frontal gyri, then, there is some evidence of atypical connectivity with the striatum, prefrontal cortex, and medial temporal areas. Evidence of reduced connectivity with dorsal and ventral lateral prefrontal cortex in Sommer et al. (2012) and Clos et al. (2014) is limited, though, by the lack of a comparison group of non-hallucinating participants with psychosis. Two studies point to increased coupling between inferior frontal and parahippocampal regions, but in different hemispheres.

### 3.2 Default mode regions

#### 3.2.1 Temporoparietal junction

Vercammen et al. (2010) compared resting functional connectivity in SzAVH+ and HC participants among seeds in the left and right temporoparietal junction (TPJ) and a variety of ROIs, including the IFG, anterior cingulate, insula, and amygdala. While reductions in connectivity were evident generally in AVH participants, the only group difference surviving correction for multiple comparisons was a reduction in connectivity between the left TPJ and right IFG. Symptom correlations for hallucination severity were also observed for links between the left TPJ and bilateral ROIs in the amygdala and anterior cingulate, such that greater severity was associated with reduced connectivity. This relationship was observed for two separate measures of hallucination severity (P3 on the PANSS and total score on the Auditory Hallucination Rating Scale; Hoffman et al., 2003) but not for positive, negative, or general symptom scores on the PANSS. Clos et al. (2014) also reported evidence of altered left TPJ connectivity in their sample of participants with AVH and psychosis. Compared to HC controls, participants with AVH showed reduced coupling between left TPJ and left angular gyrus, and this was particularly evident in those who hallucinated during scanning, suggesting both trait and state alterations to TPJ connectivity in AVH.

As in Clos et al. (2014), Vercammen et al.’s (2010) study did not include a clinical control group of participants with psychosis but not AVH. However, their evidence of symptom correlations specific to AVH, and evidence of state effects in Clos et al. (2014), partially address the concern about other psychotic factors acting as confounds. In addition, there is also some evidence of atypical TPJ connectivity in non-patient individuals with AVH: Diederen et al. (2013) observed a negative correlation between left TPJ and right IFG in control participants, but this was not evident in participants with AVH.

#### 3.2.2 Midline structures (cingulate cortex and precuneus)

Evidence of connectivity patterns in midline structures (anterior cingulate, posterior cingulate, and precuneus) has tended to come from studies employing network analysis rather than seed-based methods. Wolf et al. (2011) observed reduced connectivity strength for SzAH+ individuals in the left anterior cingulate, right posterior cingulate, and left precuneus, alongside increased connectivity in right superior frontal gyrus and middle frontal gyrus (with connectivity being defined as the voxel weighting for that region within a resting network). Connectivity in the left anterior cingulate was also negatively associated with hallucination severity, i.e. greater connectivity strength in that region was associated with reduced hallucination proneness.

This suggests that AVH may be associated with reductions in the general connectivity of anterior and posterior midline structures. However, Wolf et al. (2011) did not include a non-hallucinating patient group, which limits interpretation of their findings. Contrasting findings were evident for posterior medial cortex in Van Lutterveld et al.’s (2014) analysis of non-clinical AVH participants, which found greater connectivity strength for the right posterior cingulate and precuneus. Correlations between ACC connectivity and hallucinations have been observed in other studies, but they vary with area: increased hallucination severity has been associated with reduced coupling between bilateral ACC and left TPJ (Vercammen et al., 2010), but elevated connectivity between ACC and left PAC (Shinn et al., 2013).

#### 3.2.3 Hippocampal formation (hippocampus and parahippocampal cortex)

Four of the above studies (Clos et al., 2014; Diederen et al., 2013; Shinn et al., 2013; Sommer et al., 2012) and one other study (Rotarska-Jagiela et al., 2010) have reported specific resting differences in the hippocampus and parahippocampal cortex (PHC). Rotarska-Jagiela et al. (2010) examined resting networks in a sample of patients with schizophrenia and a history of AVH compared to an HC group. While general differences in connectivity between Sz and HC participants were widespread, hallucination severity was related to decreased connectivity in the lower part of the hippocampus and the left posterior STG. In both regions, however, connectivity reductions were also associated with delusion severity, suggesting a relationship with positive symptomatology in general, rather than hallucinations in particular.

Elsewhere, increased levels of connectivity in AVH participants have been reported between left hippocampus to left thalamus (Clos et al., 2014); left PHC to left IFG (Diederen et al., 2013); and right PHC to right IFG (Sommer et al., 2012). Decreased coupling has been observed between right PHC and left PAC (Shinn et al., 2013) and left hippocampus to left STG (Sommer et al., 2012). The strongest of these findings is provided by Sommer et al. (2012), who also observed decreased STG-hippocampus connectivity for those who reported AVH during scanning and scored higher for AVH symptoms in general.

Overall, then, findings on hippocampal connectivity in individuals with AVH are very mixed and non-replicating. Primarily there is evidence of alterations in resting connectivity among a variety of hippocampal and language regions, but this may also reflect choice of seeding regions. Only one study—Sommer et al. (2012)—has reported combined evidence of connectivity alteration, symptom correlation, and state effects.

### 3.3 Other networks and between-network interactions

#### 3.3.1 Insula and striatum

One study has specifically studied connectivity of the insulabased salience network. Manoliu et al. (2014) used ICA to compare connectivity levels within the salience network for individuals with current psychosis, individuals in remission, and healthy controls. Hallucination (but not delusion) severity was negatively associated with connectivity strength in the right anterior insula specifically. Increased functional connectivity between the left insula and left IFG was also observed in AVH participants by Clos et al. (2014), while Vercammen et al. (2010) included bilateral insula ROIs in their analysis but found no significant alterations in connectivity.

Sorg et al. (2013), using the same sample as Manoliu et al. (2014), reported on resting state connectivity within a basal ganglia network that included the striatum, globus pallidus, and the thalamus. Hallucination severity within the network was specifically associated with connectivity in the putamen. However, this relation was also observed for delusion scores. Thus, while both Sorg et al. (2013) and Manoliu et al. (2014) lacked a specific comparison of clinical participants with and without AVH, only the latter could demonstrate hallucination-specific correlations with resting connectivity.

#### 3.3.2 Interactions between the DMN and other networks

Finally, two studies have examined how interactions among different networks may contribute to AVH (Jardri et al., 2013; Manoliu et al., 2014). Jardri et al. (2013) examined neural activation during hallucinatory episodes and at rest in a group of adolescents with brief psychotic disorder, who either experienced AVH, visual hallucinations or both. Hallucination episodes were associated with greater activation in associative sensory areas specific to the modality of the hallucination (including the STS and occipito-temporal junction), and this correlated with hallucination severity. When associative sensory cortical activation increased, this anti-correlated with activity in the DMN, and hallucination severity predicted both the DMN goodness of fit (a measure of how stable the DMN was spatially) and its variability over time. Furthermore, there was no relationship observed between DMN instability and positive symptoms in general. This led Jardri and colleagues to suggest that hallucination periods may be preceded by a sudden disengagement of unstable DMN states, leading to internal representations (such as those activated by the memory functions of the DMN) being processed by as if they were external, sensory stimuli.

There is also some evidence linking AVH with the interaction between the DMN and the central executive network (CEN). In addition to analyzing the salience network, Manoliu et al. (2014) examined properties of both the DMN and CEN in their sample of participants with schizophrenia. A positive relationship was observed between hallucination severity and interconnectivity between dorsal nodes of the DMN and right ventral CEN, with no corresponding association for delusion scores. Thus, while hallucination onset may be prompted by weaker or more unstable interactions between the DMN and sensory networks, hallucination severity may be associated to tighter coupling (and by extension, less separation) between the DMN and networks serving attentional control.

## 4 Confounds to the resting state: hallucinations, medication, and movement

Observing atypical resting states in those prone to AVH raises the question of whether they should be interpreted as trait or state effects: that is, as markers of hallucination-proneness or as characteristics of the hallucinatory state itself. Most resting-state studies focus on the former, with separate symptom-capture designs being used to study the study the state of hallucination. As such, ruling out or controlling for the presence of AVH during scanning is important for understanding their contribution to resting dynamics.

Of the above studies, the majority have controlled for the state of AVH in various ways (see Table 2). Four studies only included hallucination-free data, either by excluding participants who experienced AVH during scanning (Diederen et al., 2013; Van Lutterveld et al., 2014), analyzing AVH data elsewhere (Hoffman et al., 2011), or checking that their participants reported no hallucinations (Gavrilescu et al., 2010). Four studies specifically compared data with and without AVH, either between participants (Clos et al., 2014; Shinn et al., 2013; Sommer et al., 2012) or within participants (Jardri et al., 2013). The remaining studies reported no specific attempts to control for the presence of hallucinations.

Use of antipsychotic medication is another factor that may be expected to affect the characteristics of resting state networks. Although there are only a limited of number of studies in this area, antipsychotic administration has been found to alter connectivity strength between the vmPFC and the rest of the DMN (Sambataro et al., 2009), between the mPFC and both the hippocampus and nucleus accumbens (Bolding et al., 2012) and between temporal and parietal regions, temporal and frontal regions and the precuneus and basal ganglia (Lui et al., 2010). Antipsychotics hence have the potential to act as a notable confound for existing studies of the relation between AVH and RSNs. All of the studies that included clinical participant groups reported on medication use, with the exception of Jardri et al. (2013), who specifically recruited medication-free participants. Attempts to control for medication effects were mixed: two studies reported no group differences in medication levels between clinical groups with and without AVH (Gavrilescu et al., 2010; Shinn et al., 2013), while five studies either tested for correlations with medication levels or corrected symptom correlations according to medication use (see Table 2). Wolf et al. (2011) found that connectivity abnormalities in right MTG in participants with AVH also correlated with medication use, but no other studies reported similar overlaps. Three studies either reported no correction for medication use or could not do so because of diversity in the kinds of medications used (Clos et al., 2014; Sommer et al., 2012; Vercammen et al., 2010).

Beyond the influence of AVH and medications, clinical status is also associated with other potential confounding factors. For instance, patients often move in the scanner more than controls, and this can greatly affect connectivity results for seed-based methods in particular (Van Dijk et al., 2012). Motion problems are known to affect imaging reliability in research with children (Satterthwaite et al., 2012), older adults (Mowinckel et al., 2012), and individuals high in impulsivity (Kong et al., 2014), and it is highly likely that studies with people currently experiencing psychosis will be similarly affected. This problem can be addressed to some degree by regressing out head-motion parameters and other nuisance variables (such as cardiorespiratory measures) from fMRI time series, but even this has been argued to leave considerable artifacts in the data, leading to overestimation of local connectivity and underestimation of long distance connectivity (Power et al., 2012). Use of ICA-based methods allows for the removal of movement-related components, but also involves a degree of subjective decision-making in the identification of which components to ignore. Of the above studies, six report use of standard regression methods to correct for head motion, while two used both regression and ICA (Rotarska-Jagiela et al., 2010; Van Lutterveld et al., 2014). The remainder reported no specific motion correction but many of them did use ICA, which may have included identification of motion effects. Notably, only two studies reported group comparisons for overall levels of head movement during scanning: Shinn et al. (2013) and Van Lutterveld et al. (2014).

Finally, concurrent problems with agitation or anxiety may be expected to be more common in patients compared to control participants, but of the above studies, only one (Shinn et al., 2013) reported specifically asking participants about their anxiety levels and general mood during scanning (the participants studied by Sorg and Manoliu were asked about any “odd feelings” during scanning, but more specific details are not reported). No studies have used standardized state measures of anxiety, despite the fact that it can influence resting-state characteristics (Dennis et al., 2011).

## 5 Discussion

Within a relatively short space of time (2010–2014), a number of studies have reported on alterations in intrinsic functional connectivity in individuals with AVH. The first key observation to make is that few if any findings have directly replicated across studies. Perhaps most strongly implicated is the connectivity of the left STG, but even this shows evidence of both elevated and reduced functional connectivity across different studies. That said, few studies show directly conflicting results, with the exception of IFG–STG connectivity (Hoffman et al., 2011; Sommer et al., 2012) and the results for primary auditory areas (Gavrilescu et al., 2010; Shinn et al., 2013).

The lack of replication across studies largely reflects methodological heterogeneity, rather than necessarily implying contradictory findings. The studies reviewed vary in several respects, including their use of seed-based or network methods, their selection of seed placements, their choice of ROI or whole-brain connectivity analyses, artifact corrections, and the specificity of participant groups. This variation across studies makes it a challenge to clearly distinguish reliable findings from false positives.

Nevertheless, the reviewed studies do highlight some regions and networks whose intrinsic functional connectivity is likely to be implicated in proneness to AVH. In particular, connectivity in left temporal cortex appears to be strongly involved in AVH, ranging from PAC to the superior temporal gyrus and on to the left TPJ area. Left PAC shows mixed levels of connectivity for its coupling with frontal cortex and hippocampal structures, and may also have reduced interhemispheric connectivity with right PAC, but resting-state differences are by no means confined to this area (cf. Cho and Wu, 2013). Left STG shows mostly elevated functional connectivity across seed- and network-based studies, but also reduced coupling with hippocampal and thalamic areas. There is evidence of the left TPJ showing reduced coupling with immediately surrounding structures (angular gyrus), inferior frontal cortex, and the anterior cingulate. In many cases right temporal seed regions have also been studied but have not yielded comparable alterations to connectivity, highlighting the specific importance of left temporal cortex to AVH.

Alterations to resting connectivity in left STG are consistent with evidence of associations between hallucination severity and gray matter reduction in this area (Modinos et al., 2013; Palaniyappan et al., 2012), evidence of its activation in symptom-capture studies (Jardri et al., 2011), and atypical connectivity with frontal cortex during self-monitoring tasks (Wang et al., 2011). However, evidence of atypical resting connectivity between speech production and comprehension areas is much more equivocal, in contrast to evidence of reduced structural connectivity between left STG and left IFG (Geoffroy et al., 2014; McCarthy-Jones et al., 2015). One study has indicated reduced IFG–STG synchronization (Sommer et al., 2012), while another has observed elevated connectivity, albeit along an extended loop that also contains striatal regions (Hoffman et al., 2011). Other studies that included the same or similar seed regions reported no alterations to left IFG–STG connectivity (Diederen et al., 2013; Vercammen et al., 2010). Taken together, the results for left PAC and STG suggest that both basic and higher-level speech-processing areas show altered intrinsic connectivity, but not necessarily in their coupling with speech production areas.

Alterations to resting connectivity in left TPJ and—to a more varied extent—hippocampal and midline structures implicates the default mode network in the development of AVH. The best evidence of DMN involvement is provided by Jardri et al.’s (2013) finding that DMN instability predicted hallucination severity. Weaker coupling within the DMN, as the collection of findings for left TPJ and ACC would appear to highlight, could conceivably contribute to an unstable resting network. The exception to this is the finding of greater connectivity strength in PCC/precuneus by Van Lutterveld et al. (2014), although this may be due to the use of non-clinical participants with AVH. Based on evidence of greater correlation between the DMN and other networks in those with AVH (e.g., Manoliu et al., 2014), it seems plausible that atypical modulation of the DMN in either direction (that is, a weak and unstable DMN or an overly strong and active DMN) could give rise to internal cognitions being mistakenly processed in sensory association areas, and that this may vary across clinical and non-clinical participant groups. Atypical DMN modulation is also evident in general schizophrenia groups (Whitfield-Gabrieli and Ford, 2012), however, meaning that more work is needed to delineate why hallucinations (and not other psychotic symptoms) could arise in this way.

Methodologically, most of the above studies have taken clear steps to control for the potential influence of antipsychotic medication where appropriate, with only three studies making no correction at all (Clos et al., 2014; Sommer et al., 2012; Vercammen et al., 2010). Those that sought to focus specifically on AVH (rather than reporting on a general schizophrenia group with symptom correlations) also controlled for the presence of AVH during scanning, either via excluding AVH data or including direct comparisons between participants or epochs in which hallucinations were reported. Of greater concern is the general lack of specific measures to combat motion artifacts, over and above standard nuisance regression techniques. Head motion—which is influenced by a range of individual differences, including clinical status—is thought to specifically reduce long-range connectivity indices and affect anterior–posterior networks such as the DMN or CEN (Van Dijk et al., 2012). As such, evidence of connectivity reductions for these networks in AVH participants compared to controls must be interpreted with caution in the absence of more extensive motion correction methods, such as ‘scrubbing’ of specific epochs with large artifacts (Power et al., 2012). Apparent deficits in long-range functional connectivity have been observed to largely disappear when such techniques are deployed in other clinical participant groups, such as autism (Tyszka et al., 2013); it may be fruitful to adopt similar techniques in future research with AVH samples.

One of the clearest ways to minimize such confounds is to include a non-AVH clinical comparison group alongside a healthy control group. Medication status, movement issues, and presence of other symptoms are all controlled for to a greater degree in the three studies that included both SzAVH+ and SzAVH− groups (Gavrilescu et al., 2010; Hoffman et al., 2011; Shinn et al., 2013) than the eight patient studies that did not. The inclusion of a clinical comparison group—or multiple comparison groups, such as participants with post-traumatic stress disorder, or bipolar disorder—is important for a thorough examination of what resting state abnormalities say about the specific mechanisms underlying AVH. The lack of a clinical comparison group in a number of the studies reviewed here limits their evidential value, although in most cases significant symptom correlations, specific to hallucination severity, were also reported.

Given the range of processing methods that can be deployed for measuring resting connectivity, some form of minimum analysis protocol may also be necessary for future studies on the topic, to allow for direct comparison across datasets and confidence in the reliability of findings. This might include clear reporting policies on the overall state of participants during scanning (including but not exclusive to the presence of AVH), specific measures to counter motion problems, and use of comprehensive sets of theory-driven ROIs (as in Vercammen et al., 2010, and Diederen et al., 2013, for example). These could be reported on for each resting-state study of AVH before the deployment of more complex or exploratory analysis, such as network or graph theoretical approaches. The specification of seed regions will depend on the development of good theoretical models for the development of AVH, although this could be achieved in an iterative manner, with resting state findings and neurocognitive models mutually informing one another. The specification of prospective subtypes for AVH, including a distinction between voices relating to inner speech and memory processes (McCarthy-Jones et al., 2014), and the development of cross-laboratory protocols, as advocated by the International Consortium on Hallucination Research (ICHR: Waters et al., 2012, 2014), could provide a preliminary framework for nominating candidate seed regions.

Other factors important to consider are how resting neurophysiological differences may contribute to AVH, and the relation between structural and functional connectivity alterations in those prone to hallucination. In particular, fluctuation in EEG ‘microstates’—short-lasting but stable couplings between neuronal assembles—has been linked to the occurrence of AVH (Kindler et al., 2011). How resting dynamics vary on scale of milliseconds (compared to the >1 s temporal resolution of fMRI), and how they relate to the RSNs discussed above, requires use of combined EEG/MRI methods or improved source localization methods in MEG. A key question is whether such fluctuations could underpin the apparent instability in the DMN observed by Jardri et al. (2013) for participants with hallucinations.

Resting functional connectivity also often (but not always) reflects underlying white-matter connections (Greicius et al., 2009). In the case of AVH, structural alterations may be expected to reflect trait characteristics of a network, while resting functional connectivity seem likely to reflect both trait and state characteristics. As noted above, evidence of structural reductions in white-matter integrity in the left arcuate fasciculus do not clearly map onto alterations in intrinsic functional connectivity between speech production and comprehension regions (Hoffman et al., 2011). However, two issues are worthy of consideration here. First, the failure of resting state studies to find clear evidence for functional changes between left IFG and left STG, in the context of documented structural changes to the left arcuate fasciculus, may be due to the low temporal resolution of fMRI-based functional connectivity studies. Evidence has been found that reduced structural integrity in the left arcuate fasciculus of people with schizophrenia is associated with an approximately 50 ms delay in corollary discharge signaling between frontal and temporal regions (Whitford et al., 2011), potentially due to dysmyelination or demyelination of this tract (McCarthy-Jones et al., 2015). Such subtle timing differences make assessment of functional connectivity using mixed-methods, including EEG/MEG (e.g., Brookes et al., 2011) a promising way to further explore how underlying differences in physical connectivity contribute to RSN dynamics in AVH. Second, theoretical arguments (Whitford et al., 2012) and empirical evidence (McCarthy-Jones et al., 2015) that state AVH may be non-linearly related to structural changes in the left arcuate fasciculus, suggest that exploring non-linear relations between AVH and IFG–STG functional connectivity may be fruitful.

In conclusion, initial research on intrinsic functional connectivity in AVH has highlighted a variety of regions that show altered resting properties in those prone to hallucinations, including areas implicated language and default mode networks. The strongest evidence supports altered resting connectivity in the left superior temporal gyrus, although there is evidence of connectivity alterations throughout the left temporal cortex. The field of resting state research has great potential for unmasking the underlying mechanisms of AVH and in doing so provide a clear testing ground for different theories of voice-hearing. However, comparisons across studies are limited by extensive methodological heterogeneity, suggesting that an agreed analysis pipeline, with more attempts at direct replication, may be important for future research to progress effectively and systematically.

## Figures and Tables

**Table 1:** Resting functional connectivity MRI studies in individuals with AVH.

Study	Groups	Method	Connectivity in AVH participants	Symptom correlation
Gavrilescu et al. (2010)	SzAVH+ SzAVH– HC	Seed (left and right PAC)	↓ Left PAC - right PAC	–
Shinn et al. (2013)	SzAVH+ SzAVH– HC	Seed (left and right PAC)	↑ Left PAC - left SPL, left MFG ↓ Left PAC - right PHC, right thalamus	↑ Left PAC - left IFG, left STG, right OFC, right pre- and post-central gyrus, ACC and PCC
Hoffman et al. (2011)	SzAVH+ SzAVH– HC	Seed (bilateral STG)	↑ Bilateral STG - left IFG - putamen	–
Sommer et al. (2012)	Psychosis + AVH HC	Seed (left STG and right IFG)	↑ Right IFG - right PHC ↓ Right IFG - right DPLFC ↓ Left STG - left IFG, left hippocampus	↓ Left STG - left hippocampus
Clos et al. (2014)	Psychosis + AVH HC	Seed (left IFG, MTG, AG, thalamus)	↑ Left IFG - left insula, SMA; Left hippocampus - left thalamus ↓ Left IFG - left IPL, left DLPFC, bilateral VLPFC ↓ Left MTG - right STG; left TPJ - left AG; Left thalamus - right cerebellum	↑ Left IFG – left VMPFC ↓ Thalamus – right PCG, right PHC
Diederen et al. (2013)	NC + AVH HC	Seed (bilateral IFG, STG, PHC)	↑ Left STG - right STG, right IFG; left IFG - left PHC	
Van Lutterveld et al. (2014)	NC + AVH HC	Network/ICA	↑ Left STG, right MTG, PCC (strength) ↑ Left STG (centrality) ↓ Left PCL, right ITG, right amygdala (centrality)	–
Wolf et al. (2011)	SzAVH+ HC	Network/ICA	↑ Left STG, right SFG, right MFG, bilateral MTG ↓ Left ACC, left precuneus, right PCC	↑ Left STG, right MFG ↓ Left ACC
Vercammen et al. (2010)	SzAVH+ HC	Seed (left and right TPJ)	↓ Left TPJ - right IFG	↓ Left TPJ - bilateral ACC, amygdala
Rotarska-Jagiela et al. (2010)	SZ HC	Network/ICA	–	↓ Left hippocampus, left STG
Manoliu et al. (2014)	SZ HC	Network/ICA	–	↓ Right AI ↑ DMN-CEN
Sorg et al. (2013)	SZ HC	Network/ICA	–	↓ Putamen
Jardri et al. (2013)	Brief PD HC	Network/ICA	↓ GoF in DMN, DMN–ASC	↓ DMN stability

AI = anterior insula, ACC = anterior cingulate cortex, AG = angular gyrus, ASC = association sensory cortex, AVH = auditory verbal hallucination, CEN = central executive network, DLPFC = dorsolateral prefrontal cortex, DMN = default mode network, GoF = goodness of fit, HC = healthy control, ICA = independent components analysis, IFG = inferior frontal gyrus, IPL = inferior parietal lobule, ITG = inferior temporal gyrus, MFG = middle frontal gyrus, MTG = middle temporal gyrus, NC = non-clinical, OFC = orbitofrontal cortex, PAC = primary auditory cortex, PCC = posterior cingulate cortex, PCG = precentral gyrus; PCL = paracentral lobule, PD = psychotic disorder, PHC = parahippocampal cortex, SMA = supplementary motor area, SPL = superior parietal lobule, Sz = schizophrenia, SFG = superior frontal gyrus, STG = superior temporal gyrus, TPJ = temporoparietal junction, VLPFC = ventrolateral prefrontal cortex; VMPFC = ventromedial prefrontal cortex.

**Table 2:** Control for state effects of hallucination, medication, and movement in resting state AVH studies.

Study	AVH control?	Medication control?	Motion control?
Gavrilescu et al. (2010)	Yes (no AVH data included)	Yes (no group difference)	Regression
Shinn et al. (2013)	Yes (group contrast)	Yes (no group difference)	Regression and group comparison
Hoffman et al. (2011)	Yes (no AVH data included)	Yes (correlation)	Regression
Sommer et al. (2012)	Yes (group contrast)	No	Regression
Clos et al. (2014)	Yes (group contrast)	No	Regression
Diederen et al. (2013)	Yes (no AVH data included)	N/A^a^	Regression
Van Lutterveld et al. (2014)	Yes (no AVH data included)	N/A^a^	Regression, ICA and group comparison
Wolf et al. (2011)	No	Yes (correlation)	None reported (ICA used)
Vercammen et al. (2010)	No	No	None reported
Rotarska-Jagiela et al. (2010)	No	Yes (correlation)	Regression and ICA
Manoliu et al. (2014)	No	Yes (correlation)	None reported (ICA used)
Sorg et al. (2013)	No	Yes (correlation)	None reported (ICA used)
Jardri et al. (2013)	Yes (within-subject contrast)	Yes (unmedicated)	None reported (ICA used)

AVH = auditory verbal hallucination, ICA = independent components analysis.

a Participants in Diederen et al. (2013) and Van Lutterveld et al. (2014) came from the non-clinical population.
